# Microbiome Comparison and Pathogen Identification for Three Migrating Passerines Captured During Spring Season in Jordan Using 16S rRNA Sequencing

**DOI:** 10.1093/icb/icaf015

**Published:** 2025-04-16

**Authors:** Nisreen Al-Hmoud, Mu'men Alrwashdeh, Fares Khoury, Amani Abdien, Ahmad Hayek, Ghadeer Alzghoul, Ahmad Islaieh, Cally E Erickson, Andrew W Bartlow, Jennifer C Owen, Jeanne M Fair

**Affiliations:** Bio-Safety and Bio-Security Center, Royal Scientific Society, Amman 11941, Jordan; Graduate Studies Council, Princess Sumaya University for Technology, Amman 11941, Jordan; Bio-Safety and Bio-Security Center, Royal Scientific Society, Amman 11941, Jordan; Department of Biology and Biotechnology, American University of Madaba, Madaba 11821, Jordan; Bio-Safety and Bio-Security Center, Royal Scientific Society, Amman 11941, Jordan; Bio-Safety and Bio-Security Center, Royal Scientific Society, Amman 11941, Jordan; Bio-Safety and Bio-Security Center, Royal Scientific Society, Amman 11941, Jordan; Department of Biology and Biotechnology, American University of Madaba, Madaba 11821, Jordan; Department of Genomics and Bioanalytics, Los Alamos National Laboratory, Los Alamos, NM 87545, USA; Department of Genomics and Bioanalytics, Los Alamos National Laboratory, Los Alamos, NM 87545, USA; Department of Fisheries and Wildlife, Michigan State University, East Lansing, MI 48824, USA; Department of Modeling and Observations of Earth Systems, Los Alamos National Laboratory, Los Alamos, NM 87545, USA

## Abstract

Jordan is located on an important spot along the Mediterranean and Black Sea Flyway. Hundreds of migratory bird species have been identified stopping over in Jordan during spring and autumn migratory seasons. Compared to mammals and economically important birds, the microbiomes of wild bird species are severely understudied. Gut microbial composition is a valuable source of information that reflects food preferences, foraging behavior, and the risk of pathogen transmission to humans and other animals. In this study, we assessed the microbiome composition of three species of migrating passerines (willow warblers, lesser whitethroats, and common reed warblers) captured during the spring migration stopover in Jordan in 2023. A total of 59 fecal samples were selected evenly from the three species and subjected to 16S sequencing and microbiome analysis. Our objectives were to determine the diversity of bacteria in these three species, assess the amount of intra- and inter-specific variation, and detect pathogenic genera and species that could pose health risks to humans, domestic animals, and wildlife. Bacteria mainly belonged to the phyla Proteobacteria (62%), Actinobacteriota (18%), Firmicutes (13%), Cyanobacteria (5%), and Bacteroidota (1%). The results reveal that lesser whitethroats had the greatest variation in bacterial genus richness, Shannon diversity, and microbial composition compared to willow warblers and common reed warblers. The three bird species harbored several pathogenic genera and species, including *Campylobacter, Enterococcus, Escherichia-Shigella, Mycoplasma, Rickettsia, Clostridium perfringens*, and *Vibrio cholerae*. We suggest further investigation to understand the relationship between migratory behavior and their gut microbiome. We advocate for the use of advanced molecular techniques to characterize the pathogens found in migratory birds that might have public and environmental health impacts in addition to economic loss.

## Introduction

The gut microbiome is the collection of microbes, including bacteria, archaea, fungi, protozoa, and viruses living in the intestinal tract and plays important roles in host physiology and health (Kohl 2012a). The formation and maintenance of the gut microbiome and its impact on host fitness have been the subject of numerous investigations (Rooks et al. 2014; Waite and Taylor 2015; Grond et al. 2018). Research on the structure and composition of vertebrate gut microbiota communities as well as their interactions with their hosts has advanced quickly during the past 10 years (Ley et al. 2008; McFall-Ngai et al. 2013). Animals’ immune systems, metabolism, and food digestion are all known to be significantly influenced by their gut microbiota (Flint et al. 2012; Nicholson et al. 2012). Due to their unique diets, immune systems, physiological traits, and high-energy needs (Kohl 2012b), birds have a very different gut microbiota than other animals (Hird et al. 2014; Waite and Taylor 2014, 2015). Studies on avian microbiomes have mainly focused on either economically important birds, such as chickens and turkeys, or rare wild species (Jacobs et al. 2019; Bartlow et al. 2022; Deryabin et al. 2024).

The microbial gut communities of wild birds have received some attention because they can harbor a myriad of pathogens that can affect the health of humans and wild and domestic animals (Gong et al. 2008; Thomas et al. 2008; Tsiodras et al. 2008; Garcia-Mazcorro et al. 2017; Wilkinson et al. 2017; Li et al. 2018; Wang et al. 2018, 2020; Owen et al. 2021b; Goa 2022; Sun et al. 2022). At a stopover site in Sicily, Italy, passerines had *Salmonella bongori, Yersinia enterocolitica*, and *Klebsiella pneumonia*, among others, including those bacteria resistant to antibiotics (Foti et al. 2011). Additionally, they also harbor ticks, which can be transported to new locations during migration. These ticks, and the birds, can be infected with tick-borne pathogens, such as *Rickettsia* spp. and *Anaplasma* spp. (Hornok et al. 2014; Mărcuţan et al. 2016). However, these microbiome studies are outnumbered by those on wild mammals and domestic animals. There is evidence implicating migratory birds in the intercontinental dispersal of zoonotic pathogens (pathogens transmitted from animals to humans), including Arboviruses, influenza viruses, and bacterial pathogens (Jankowski et al. 2013; Dolinski et al. 2020, 2022; Owen et al. 2021a). A bird’s capacity to serve as a reservoir and lead to transmission events of zoonotic pathogens is driven by many internal (e.g., host physiology and genetics) and external (e.g., environmental stressors) factors. In mammals, the microbiome has been linked to the pathogen loads or the capacity to transmit pathogens; yet, this linkage has not been fully investigated in wild birds and may be an important determinant of a bird’s susceptibility to pathogens and capacity to serve as a reservoir (Sun et al. 2022).

Gut bacteria primarily derive their nutrients from carbohydrates that host birds consume, resulting in metabolites that support various physiological functions, including maintenance of the gut barrier and immunocompetence (Belkaid and Hand 2014; Kogut and Fernandez-Miyakawa 2022). The symbiotic relationship between gut microbiota and their host can be impacted in many ways, resulting in dysbiosis, or an abnormal composition of bacteria species colonizing the gut. This can be detrimental to the host (Ottinger et al. 2024) and may lead to more pathogenic bacteria propagating in the gut and being released in the environment. In particular, the stress and energy requirements from migration can impact the microbiome of migrating birds and lead to more pathogenic bacteria (Obrochta et al. 2022; Thie et al. 2022; Włodarczyk et al. 2024).

The gut microbiota may vary according to (a) intrinsic factors, namely species (host genetics), sex, and age, and (b) extrinsic factors such as diet, environment, migration, and social interactions (Sun et al. 2022). Investigating temporal patterns and the impact of local environmental factors and food sources on the gut microbiotas of wild passerines may be particularly important during migration. Since most birds are unable to fly continuously between their wintering and breeding grounds, they occasionally pause their flight to refuel at stopover sites (Newton 2023). The composition of bacteria linked to the extreme physiological challenge of migration is the primary focus of the present research on the gut microbiome of migratory passerines.

The implementation of 16S rRNA next-generation sequencing (NGS) has revolutionized avian microbiome research, allowing for precise analysis of microbial diversity and community dynamics on the hypervariable region in 16S ribosomal RNA genes to identify each bacterial species from the sample (Satam et al. 2023). The high-throughput capability of NGS supports extensive comparisons across bird populations, species, and ecological contexts. By sequencing the variable regions of the 16S rRNA gene, this method provides a detailed profile of gut microbiota, making it a powerful tool for identifying pathogens and examining microbial diversity across diverse hosts and environments (Klindworth et al. 2013).

In this study, we assessed the microbiome composition of three species of long-distance migrating passerines captured during a migration stopover in Jordan. Our study had three main objectives: (1) determine the diversity of bacteria in fecal samples; (2) assess the amount of intra- and inter-specific variation in composition of the gut microbiota; and (3) screen the microbiome for pathogenic genera and species that could pose health risks to humans, domestic animals, and non-avian wildlife. To our knowledge, this is the first use of 16S sequencing to characterize the microbiome and detect pathogens in migratory passerines in Jordan.

## Materials and methods

### IACUC approval and governmental permissions

An Institutional Animal Care and Use Committee (IACUC) approval was obtained for this study within the context of the international collaborative project “The Mediterranean and Black Sea Flyway: Transboundary Determinants of Avian Zoonotic Infectious Diseases Avian Zoonotic Disease Network (AZDN)” (Fair et al. 2024) for using, handling, and sampling birds from the IACUC committee at the College of Veterinary Medicine of Jordan University of Science & Technology (Irbid-Jordan), number (16/4/12/420) dated on August 23, 2022.

Two official permissions were obtained; the first one was from the Ministry of Environment to implement the project’s activities in Jordan (Permission No. 3/5/2811 dated on March 29, 2022). The second one was obtained from the Royal Society for Conservation of Nature “RSCN” (Amman-Jordan) to access their reserves throughout Jordan to capture, handle, and sample migratory birds (Permission No. GM/15/1/454 dated on May 23, 2022).

### Azraq wetland reserve

Jordan is located on one of the main routes for bird migration; the Mediterranean-Black Sea Flyway (Andrews 1995). According to Jordan BirdWatch (https://www.jordanbirdwatch.com/birds-in-jordan/jordan-bird-list/) 440 bird species have been recorded in Jordan of which around 95 are year-round residents. Most of the remaining species are migratory and either winter or breed in Jordan, or are passage migrants that temporarily stopover in Jordan while migrating between their northern breeding and southern non-breeding areas. Jordan’s Azraq Wetland Reserve is the largest wetland in Jordan, located in the Eastern Desert (35.23°E, 29.55°N) 110 km away from the capital Amman. The Azraq Wetland Reserve is a preservation project by the RSCN. Announced in 1978, the RSCN established the Azraq Wetland Reserve for the conservation of the wetland and the migratory birds in the area.

Given its location, Al Azraq serves as an important stopover site for birds migrating along the Mediterranean-Black Sea flyway. The RSCN recorded around 300 species of resident and migrant birds from Europe to Africa that use Azraq Wetlands during the winter season (Andrews 1995). The oasis, actually a marshland fed by springs, dried out due to over pumping of ground water in the 1980s, but since 1995, the conservation efforts of RSCN restored over 10% of the original marshland area, including permanent ponds. The passerines considered in this study were captured in this marshland habitat consisting of a mix of reed, sedge (*Phragmites* and *Typha* spp.), and *Tamarix jordanis* shrubs.

### Bird species

We selected the three most common migratory land bird species trapped during April–early May 2023 at Al Azraq for microbiome analysis. The three warbler species are long-distance, Palearctic migrants that cross the Middle East during their passage to their wintering grounds in sub-Saharan Africa and the Sudan zone. The willow warbler (*Phylloscopus trochilus*) is a common summer visitor across the Palearctic region and spends the winter in central to southern Africa and parts of Asia. During the breeding season, willow warblers inhabit woodlands, including open and disturbed woods, whereas in winter they occupy savannah forests, which are nevertheless structurally similar to the breeding habitats (Lerche‐Jørgensen et al. 2019).

Most populations of the European reed warbler (*Acrocephalus scirpaceus*) are long distance migrants. The species is clearly associated with marshes and reed beds in its Palearctic breeding grounds. However, in its sub-Saharan African winter grounds, the species is often found in more arid habitats, mainly in dense scrub and grasslands, occasionally in reed beds, and locally in coastal mangroves (Dowsett-Lemaire and Dowsett 1987; Zwarts et al. 2016).

All European and Middle Eastern populations of lesser whitethroat (*Curruca curruca*) migrate *via* the Middle East to Eastern Africa in the fall season and vice versa in spring migration. Lesser whitethroat is one of the most abundant migratory land birds in Jordan (Andrews 1995; Khoury 2004). The wintering grounds are in northeast Africa, from southern Egypt to Ethiopia, Sudan, and Chad. During the breeding season, they are found primarily in open and semi-open mixed forests/forest edges, areas with low scrub, groups of dense bushes, hedgerows, or small trees, especially conifers, with dense foliage at lower levels.

### Bird capturing

We captured land birds using ten mist nets (ECOTONE, Poland; Netting (70/2) 2.5 m Height × 12 m Length, mesh size 16 × 16 mm) set up within the Azraq Wetland Reserve in areas dominated by *T. jordanis* shrubs adjacent to a freshwater pond and reedbeds. Capturing occurred intermittently with 3–5 days per sampling trip between April 8 and May 21, 2023. We operated nets for 3–4 h starting at 1 h before sunrise and checked nets at 45 min intervals. The nets were closed after up to 4–5 h of netting and operations. Mist-net technicians extracted birds and placed them in clean, cotton bags until they were processed within 1 h of capture.

### Sampling of captured birds

Once identified to species, each bird received a uniquely numbered ring (supplementary material). The birds were sexed and aged if possible, and their health condition assessed. This included muscle score, fat score, and body weight to the nearest 0.1 g using a digital balance. Muscle score was estimates using a range from 0 to 3 according to Bairlein (1995). The fat scores, or size of visible, subcutaneous fat depots were determined visually and classified according to the 9-grade scale (0–8) (Bairlein 1992). Wing and tail length were also measured. In some cases, small blood samples were taken from the brachial nerve to produce blood smears. The birds were also checked for ectoparasites, namely ticks on the head, ears, and axial regions.

Non-lethal sampling was done for each bird to analyze the microbiome. Fresh fecal samples were collected from the bird’s cotton bag using sterile cotton swabs. Cleaned bags were used each trip. The bags were disinfected after releasing the bird. By the end of each trip, the used bags were soaked overnight in (Virkon® S disinfectant and virucide) (LANXESS Co., Germany). Up to 0.2 g of feces was immersed in 500 µL of RNA*later* inside cryovial tubes (swabs were not added to the tubes), which were preserved in liquid nitrogen, transported to the Bio-Safety & Bio-Security Centre’s lab (Amman-Jordan), and stored in a −80°C freezer until subsequent analysis.

### DNA extraction

Bacterial DNA was extracted using the Zymo Research Quick DNA Fungal/Bacterial Miniprep kit (Cat. No. D6005) according to the manufacturer instructions in a biosafety cabinet class II type A2. The frozen samples were thawed at room temperature and briefly vortexed. Then, 200 µL of the sample was added to 750 µL BashingBead lysis buffer in the ZR BashingBeads lysis tube. The tubes were placed in the bead beater and processed at 15 Hz for 15–20 min, instead of the recommended 5 min at maximum speed. The ZR BashingBead Lysis tubes were centrifuged at 10,000 × *g* for 1 min, then 400 µL of the supernatant were transferred to Zymo-Spin III-F Filter in a collection tube and centrifuged at 8000 × *g* for 1 min. A total of 1200 µL of Genomic Lysis Buffer was added directly to the filtrate in the collection tube from the previous step. Then, 800 µL of the mixture from the previous step transferred to Zymo-Spin IIICR column in a collection tube and centrifuged at 10,000 × *g* for 1 min. The flow through was discarded from the collection tube, and the step was repeated. DNA Pre-wash Buffer was added (200 µL) to the Zymo-Spin IICR Column in a new collection tube and centrifuged at 100,000 × *g* for 1 min. Then, 500 µL of g-DNA Wash Buffer was added to the Zymo-Spin IICR Column and centrifuged at 100,000 × *g* for 1 min. The Zymo-Spin IICR Column was transferred to a clean 1.5 mL microcentrifuge, and 100 µL of DNA Elution Buffer was added directly to the column matrix. The Zymo-Spin IICR Column then centrifuged at 100,000 × *g* for 30 s to elute the DNA. The eluted DNA (100 µL) was stored at −80°C freezer until microbiome analysis.

### Targeted 16S microbiome analysis

The extracted DNA from the fecal samples of birds was subjected to 16S microbiome analysis at Zymo Research Co. (Germany). Briefly, the V3-V4 region of the 16S ribosomal gene was targeted for this analysis. The DNA samples were prepared using Quick-16S™ NGS Library Prep Kit (Zymo Research, Irvine, CA) using the Quick-16S Primer Set V3-V4 (cat #: D6405-2-400). The library preparation was performed once for each individual sample. Specifically, the sequencing library was prepared using real-time PCR assays, then quantified in a quantitative fluorescence PCR, and pooled together with considering their final concentrations to be equalized in the final PCR product. The sequence library was cleaned up with the Select-a-Size DNA Clean & Concentrator™ (Zymo Research, CA, USA), then quantified with TapeStation® (Agilent Technologies, CA, USA) and Qubit® (Thermo Fisher Scientific, WA, USA). The ZymoBIOMICS® Microbial Community DNA Standard (Zymo Research, CA, USA) was used as a positive control for each targeted library preparation. Blank extraction controls and blank library preparation controls were included as negative controls to assess the level of contamination generated in the lab. Finally, the prepared library was sequenced using Illumina® NextSeq™ 1000 with a P1 reagent kit (600 cycles) (Illumina Inc., CA, USA) to generate 2 × 300 bp reads. The 40% PhiX spike-in was performed for improving the run performance.

### Bioinformatics and statistical analyses

Bioinformatics for the obtained data were done using QIIME2 (version 2023.5) of the EDGE Bioinformatics website (www.edgebioinformatics.org; LANL, NM, USA) for data analysis and visualization, while R software (R Core Team 2024; version 4.3.2) (Core 2019) for statistical computing and graphics of the resulted data. The 16S reads were analyzed using QIIME2 with the Silva V3-V4 database and the DADA2 pipeline. The DADA2 pipeline performs quality control, denoises quality reads, and filters out chimeras. We used the following parameters in DADA2: Trim 5′ end Forward = 20, Trim 5′ end Reverse = 20, Truncation Len Forward = 0, Truncation Len Reverse = 0, SE Trim Length = 20, SE Truncation Len = 0. After quality control and filtering, reads were grouped into amplicon sequence variants (ASVs) reads and were further filtered and analyzed in R (R Core Team 2024; version 4.3.2) using the phyloseq package (McMurdie and Holmes 2013; version 1.46.0).

There was virtually no amplification in the negative control. We had 0.00002 ng/µL of DNA compared to 13 ng/µL in our positive control. Because of this and the fact that fecal samples have high microbial biomass, we did not attempt to bioinformatically remove reads to avoid unintentionally removing important reads and taxa (Eisenhofer et al. 2019). We filtered out ASVs that were identified as Eukaryota, Archaea, and “unassigned.” The majority of ASVs were not classified to the species level, so we used tax_glom from the phyloseq package to aggregate ASVs to the genus level. We filtered out genera that were listed as “uncultured.” We determined the proportion of bacterial phyla in each sample and grouped them according to bird species. To assess alpha diversity between the three bird species, we compared observed genus richness (number of bacterial genera identified in each sample) and the Shannon diversity index (measure of the number of genera and the relative abundance of each), without rarefying the data. Using Levene’s test, we found that variances of richness differed among bird species (*F* = 4.42, *P* = 0.017). Likewise, Shannon diversity lacked a homogeneity of variance, as shown by Levene’s test (*F* = 4.41, *P* = 0.017). Therefore, for comparisons among and between bird species, we used the Kruskal–Wallis test followed by post-hoc Dunn’s test for multiple comparisons using Bonferroni corrected *P*-values.

We compared the bacterial species composition of each bird species using non-metric multidimensional scaling (NMDS), with Bray–Curtis dissimilarly measure, *K* = 2 dimensions, and relative abundance data. Bray–Curtis dissimilarly is used to quantify the similarity in taxa composition between two sites (measure of 0 means they share the same taxa, while 1 means they do not share any taxa). We used PERMANOVAs to test for comparisons among the bird species using the adonis2 function in the vegan package (Oksanen et al. 2022; version 2.6.4) using by = “terms” and permutations = 1000. For comparisons of differences in dispersion (i.e., variation) among the three bird species, we used the betadisper function in the vegan package and the ANOVA function in the vegan package using 999 permutations.

Additionally, we searched our 16S bacterial species data for other select agents and pathogenic taxa of interest and recorded the number of reads in each sample that contained those pathogens. These pathogenic genera were selected based on pathogens that have been found in passerines, including enteric pathogens and potentially zoonotic bacteria (Foti et al. 2011; Lewis et al. 2016; Najdenski et al. 2018). We searched for the following genera in our dataset: *Stenotrophomonas, Bacillus, Staphylococcus, Mycoplasma, Ralstonia, Clostridium, Rickettsia, Pseudomonas, Streptococcus, Enterococcus, Escherichia-Shigella, Coxiella, Brucella, Burkholderia, Francisella, Yersinia, Anaplasma, Borrelia, Ehrlichia, Bartonella, Klebsiella, Aeromonas, Citrobacter, Serratia, Listeria, Lactobacillus, Vibrio, Pasteurella, Buttiauxella, Hafnia, Raoultella*, and *Monocytogenes*. We made a dataset of ASVs from these genera to create a heatmap of their abundance in each sample, using NMDS with Bray–Curtis dissimilarity. This was done with the plot_heatmap function in the phyloseq package. For these select genera, we search for any reads that were identified to the species level.

## Results

### Bird species

We captured and sampled a total of 81 common reed warblers, 76 lesser whitethroats, and 30 willow warblers. All captured birds were in a healthy status. Ticks were collected from two of the common reed warblers only. All species are sexually monomorphic; hence, sex could not be determined. Also, ages were not identifiable for any of the birds. Out of all the samples collected, 59 fecal samples were randomly selected from the three species to characterize the diversity of their microbiome.

The 59 samples (20 lesser whitethroats, 20 common reed warblers, and 19 willow warblers) produced 15,246,700 raw reads (SRA data available at the NCBI platform and referenced as PRJNA1236739 (accession numbers: SAMN47402528 to SAMN47402586)). From these reads and using DADA2, we identified 3396 ASVs. No reads were identified as Eukaryota. After removing reads from Archaea (28,245 reads) and “unassigned” (414 reads), we were left with 15,218,041 reads and 1428 taxa. Collapsing ASVs to the genus level and removing “uncultured” ASVs, there were 14,899,280 reads and 248 genera. The number of reads per sample ranged from 146,811 to 341,235 reads, with a mean of 252,530 ± 35,061 reads.

**Fig. 1:**
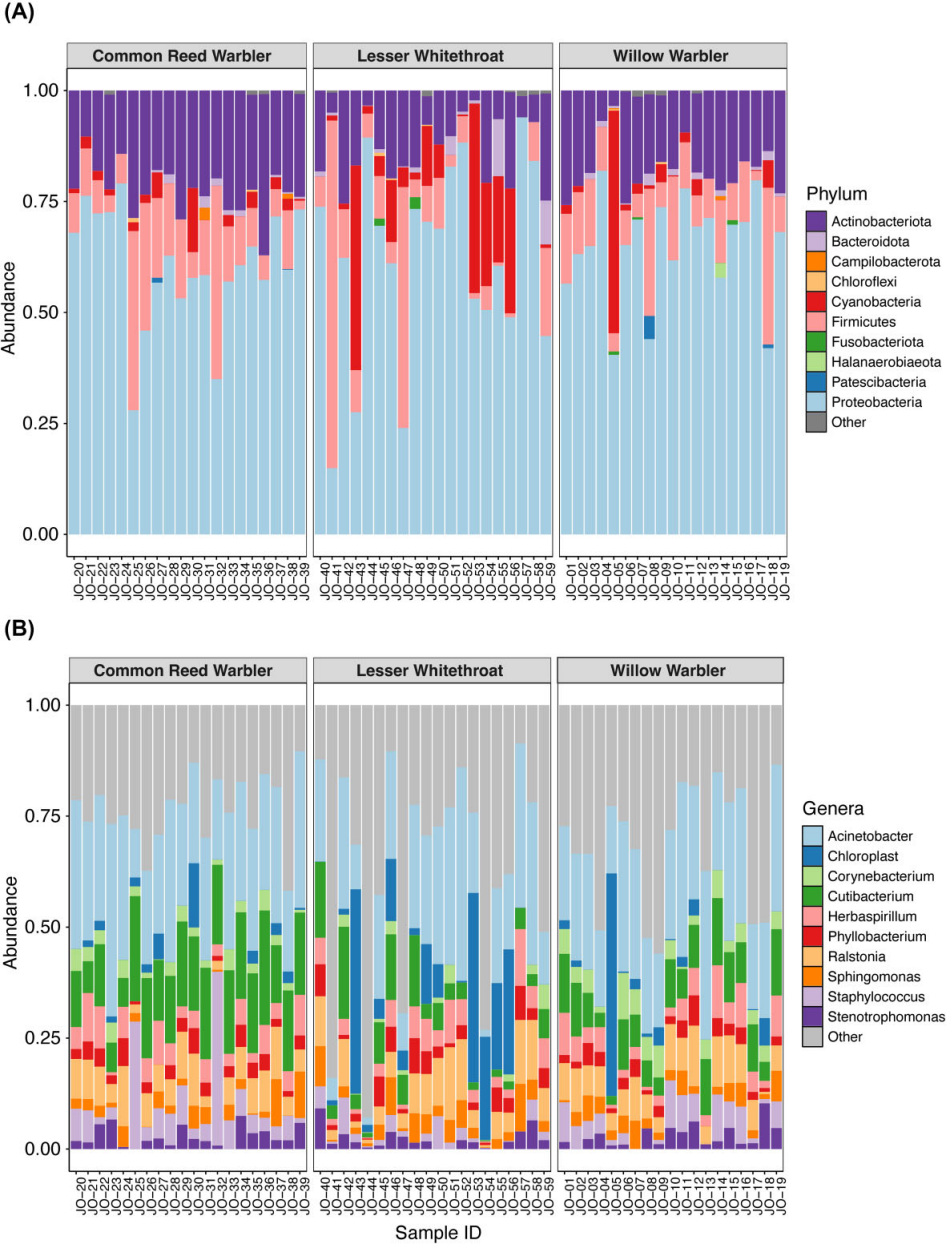
Relative abundance of the top 10 bacterial phyla (A) and genera (B) found in the microbiomes of three passerine species sampled in Jordan’s Azraq wetlands. Phyla and genera not in the top 10 were grouped into “Other.”

We compared the bacterial phyla in each sample separated by bird species. Proteobacteria (62%), Actinobacteriota (18%), Firmicutes (13%), Cyanobacteria (5%), and Bacteroidota (1%) made up the majority of phyla present in the samples. The rest of the top 10 phyla included Patescibacteria, Fusobacteriota, Campilobacterota, Chloroflexi, and Halanaerobiaeota (Fig. 1A). Phyla outside the top 10 were, in descending order, Verrucomicrobiota, Spirochaetota, Deinococcota, Gemmatimonadota, Elusimicrobiota, Desulfobacterota, Acidobacteriota, and Myxococcota. We show the relative abundance of the top 10 bacterial phyla in our samples (Fig. 1A) as well as the top 10 bacterial genera (Fig. 1B) separated by bird species.

**Fig. 2:**
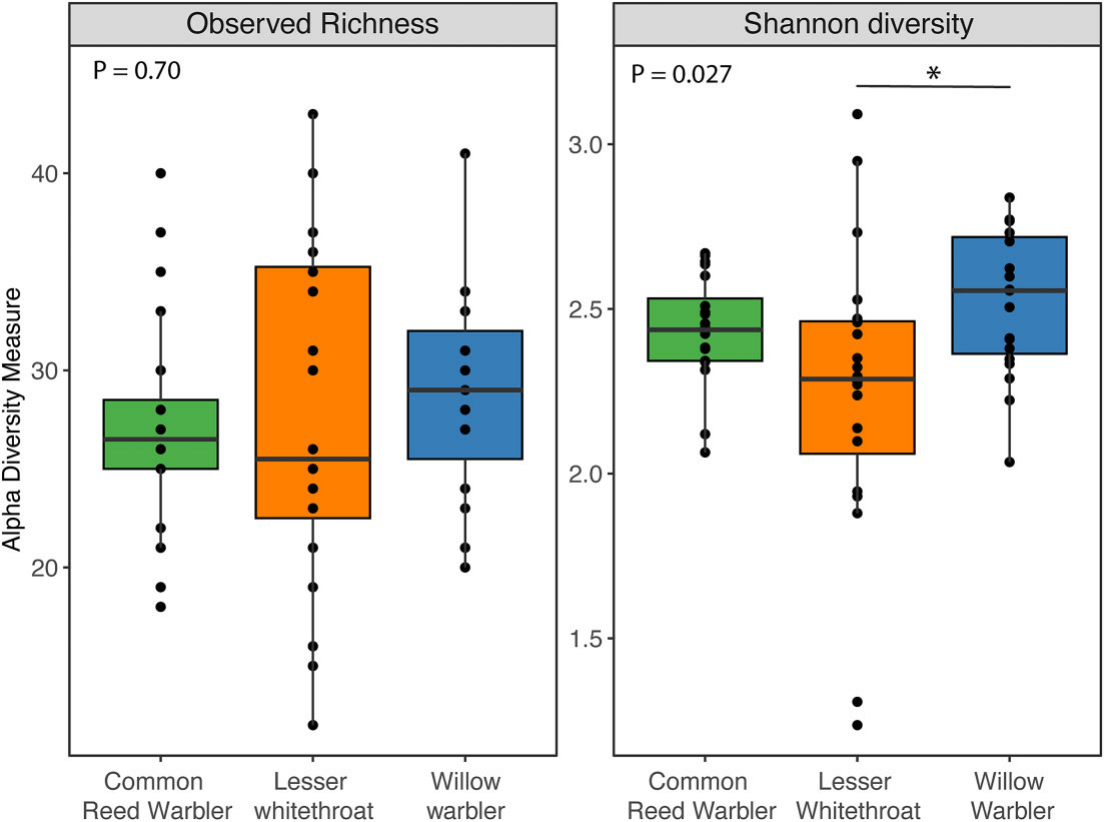
Boxplots of the observed genus richness (left) and Shannon diversity index (right) of the microbiomes from three bird species from Azraq wetlands in Jordan. Black points represent the diversity measure of each sample. Willow warblers had significantly higher Shannon diversity than the lesser whitethroat. For both measures of alpha diversity, lesser whitethroats had significantly more variation in their microbiomes than the other two species.

Bacterial genus richness was not significantly different among the three species (Kruskal–Wallis: Chi-squared = 0.718, *P* = 0.698; Fig. 2). The median and mean richness values for the three species were as follows: common reed warblers (mean: 27.3, median: 26.5), lesser whitethroats (mean: 27.5, median: 25.5), and willow warblers (mean: 28.7, median: 29.0).

Shannon diversity significantly differed among the three species (Chi-squared = 7.237, *P* = 0.027; Fig. 2). Post-hoc comparisons using Dunn’s test with a Bonferroni correction showed that the Shannon diversity index for willow warblers was significantly higher than that of the lesser whitethroats (Kruskal–Wallis: Dunn’s test: *P* = 0.025; Fig. 2). There were no significant differences in Shannon diversity between the willow warbler and the common reed warbler (Dunn’s test: *P* = 1.0; Fig. 2) or between the lesser whitethroat and the common reed warbler (Dunn’s test: *P* = 0.241; Fig. 2). The median and mean Shannon diversity values for the three species were as follows: common reed warblers (mean: 2.44, median: 2.44), lesser whitethroats (mean: 2.24, median: 2.39), and willow warblers (mean: 2.52, median: 2.56).

Using Levene’s test, we found that the genus richness within the gut microbiome differed depending on bird species (*F* = 4.42, *P* = 0.017). We used the pairwise.var.test function in the “RVAideMemoire” package version 0.9-83-7 (Herve 2022) to test for differences in variances of the pairwise comparisons using Bonferroni corrected *P*-values. The lesser whitethroats (variance = 78.5) had 185% higher variance than the willow warblers (variance = 27.5) (*P* = 0.091) and 147% higher variance than the common reed warblers (variance = 31.8) (*P* = 0.167), but these were not significant. The common reed warblers and willow warblers showed similar variances in richness (*P* = 1.0).

Shannon diversity also significantly differed in variance among the three bird species (*P* = 0.017). Again, the lesser whitethroats (variance = 0.207) showed greater variance, with 315% higher variance in Shannon diversity measure than the willow warblers (variance = 0.048) (*P* = 0.004) and 644% higher variance than the common reed warblers (variance = 0.027) (*P* > 0.001). The observed difference in Shannon diversity variation was largely due to three lesser whitethroats that were outside the quartiles. Like richness, the variation in Shannon diversity values was similar between the common reed warblers and willow warblers (*P* = 0.645).

**Fig. 3:**
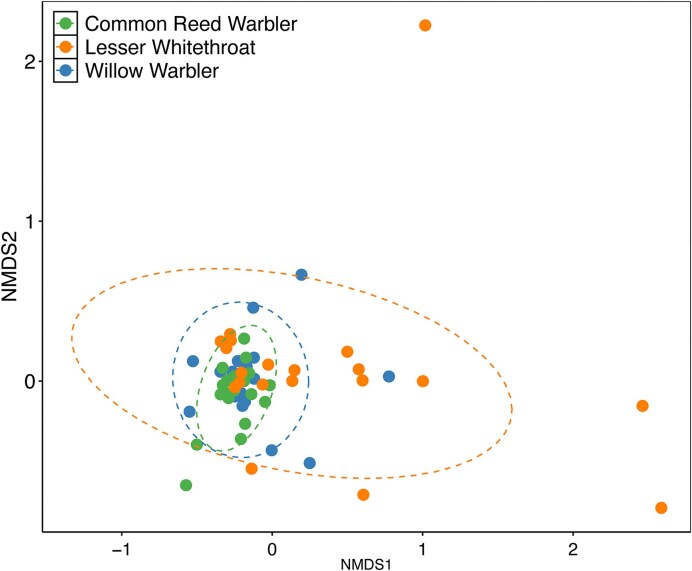
NMDS of the microbiomes from three bird species from Azraq wetlands in Jordan. Dotted ellipses represent the 95% confidence intervals of the community composition of the three bird species. The three species differed in species composition, and the lesser whitethroat had significantly more variation than the other two species.

For species composition comparisons, we used NMDS with *K* = 2 dimensions. The final stress was 0.16. The bird species significantly differed in bacterial species composition (adonis: *R*^2^ = 0.10, *P* = 0.001; Fig. 3). We then ran adonis pairwise comparisons using Bonferroni corrected *P*-values to see which species differed, and we found that each species had their own unique microbiome composition. Willow warblers significantly differed from lesser whitethroats (adonis: *R*^2^ = 0.011, *P* = 0.033) and common reed warblers (adonis: *R*^2^ = 0.052, *P* = 0.012). Lesser whitethroats and common reed warblers also differed from each other (adonis: *R*^2^ = 0.108, *P* = 0.003).

We also tested for differences in dispersion (variation) in microbial community composition since differences in dispersion may lead to differences in composition. Dispersion differed significantly between the three bird species (Betadisper: *F* = 6.916, *P* = 0.002). The lesser whitethroats had the greatest amount of variation, as assessed by the average distance to the median (0.42). The common reed warbler had the least amount of variation (0.27), and the willow warbler’s average distance was in the middle of the other two species (0.32). Compared to each other using post-hoc tests, the lesser whitethroats had greater variation in their microbial community than both the willow warblers (*P* = 0.05) and the common reed warblers (*P* = 0.002). The willow warblers and common reed warblers had similar levels of variation (*P* = 0.45) (Fig. 3).

**Fig. 4:**
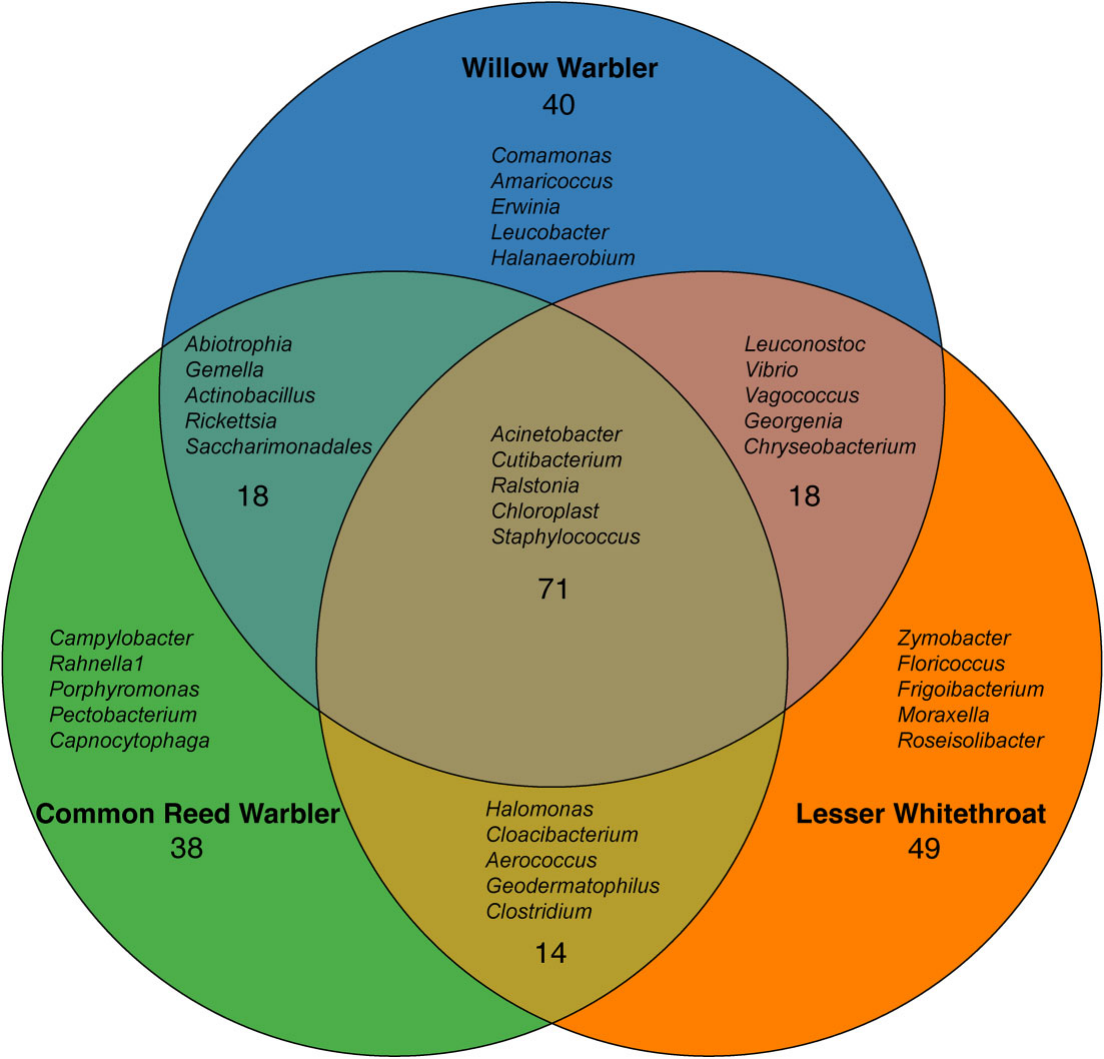
Venn diagram showing the number of shared and unique ASVs found in common reed warblers, lesser whitethroats, and willow warblers. The top five genera are listed for each combination of species and for each species separately.

A Venn diagram shows the shared and unique ASVs in each of the bird species (Fig. 4). There were 71 ASVs shared among all three bird species and 38, 49, and 40 unique ASVs in common reed warblers, lesser whitethroats, and willow warblers, respectively (Fig. 4).

**Fig. 5:**
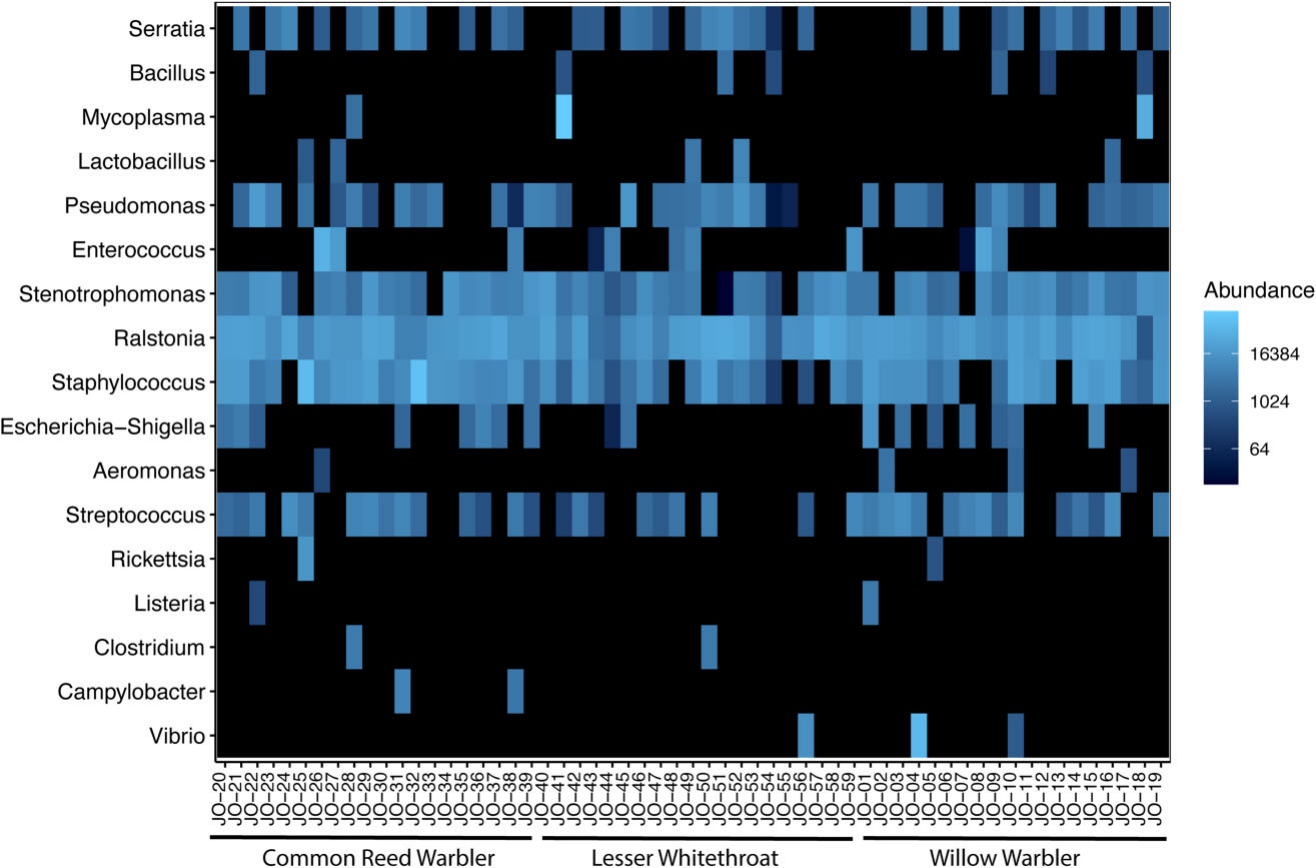
A heatmap of the species in the select pathogenic genera found in the microbiomes from three bird species from Azraq wetlands in Jordan. The scale is the abundance of reads in each sample using a log-4 transformation to better visualize the large range of values. The highest abundance of reads within a single sample is 204,760. Samples are arranged and labeled according to bird species.

A heatmap showing the abundance of potentially pathogenic genera is shown in Fig. 5, with those identified to the species level shown in Table 1. We found 17 genera consisting of 3300,825 reads. Out of the 17 genera found in our samples, there were 28 species (239,160 reads) that were identified. Four others were listed as either uncultured, from a metagenome, or an endosymbiont (*Rickettsia*). The select genera and species and the number of reads of each are listed in Table 1. The pathogens of note are *Ralstonia insidiosa, Enterococcus cecorum, Clostridium perfringens*, and *Vibrio cholerae. Ralstonia insidiosa* was found in one common reed warbler, *E. cecorum* was found in one common reed warbler, and *C. perfringens* was found in one lesser whitethroat (Table 1). We also determined the mean number of reads of each bacterial genus found in each bird species (Table 2). Most genera were found in all three bird species.

**Table 1:** Select genera and species detected from the three bird species combined using 16S sequencing.

Family	Genus	# reads per genus	Species	# reads per species
Aeromonadaceae	*Aeromonas*	5575	NA	NA
Bacillaceae	*Bacillus*	7752	*Bacillus alcalophilus*	1454
			*Bacillus circulans*	321
			*Bacillus selenatarsenatis*	2876
Campylobacteracea	*Campylobacter*	10,579	*Campylobacter gracilis*	3556
Clostridiaceae	*Clostridium*	9231	*Metagenome*	4798
			*Clostridium perfringens*	4433
Enterococcaceae	*Enterococcus*	160,159	*Enterococcus cecorum*	16,130
Enterobacteriaceae	*Escherichia-Shigella*	60,571	*Streptomyces_sp*.	4
Lactobacillaceae	*Lactobacillus*	15,835	*Lactobacillus delbrueckii*	4653
			*Lactobacillus iners*	7550
			*Lactobacillus sakei*	2023
Listeriaceae	*Listeria*	4513	NA	NA
Mycoplasmataceae	*Mycoplasma*	242,227	*Mesomycoplasma moatsii*	46,836
Pseudomonadaceae	*Pseudomonas*	165,146	*Pseudomonas brenneri*	1065
			*Pseudomonas luteola*	4434
			*Pseudomonas stutzeri*	35,068
Burkholderiaceae	*Ralstonia*	1107,087	*Ralstonia insidiosa*	12,293
			*Ralstonia pickettii*	1629
			*Uncultured beta*	7
Rickettsiaceae	*Rickettsia*	16,884	*Rickettsia endosymbiont*	646
Enterobacterales	*Serratia*	105,142	NA	NA
Staphylococcaceae	*Staphylococcus*	754,433	*Staphylococcus auricularis*	3960
			*Staphylococcus equorum*	18,864
			*Staphylococcus lugdunensis*	6711
			*Staphylococcus pasteuri*	12,974
			*Staphylococcus saprophyticus*	6534
			*Staphylococcus sciuri*	3625
			*Staphylococcus succinus*	6321
Xanthomonadaceae	*Stenotrophomonas*	378,826	*Stenotrophomonas rhizophila*	3047
			Uncultured organism	108
Streptococcaceae	*Streptococcus*	162,578	*Streptococcus gordonii*	4166
			*Streptococcus salivarius*	6839
			*Streptococcus sanguinis*	12,873
Vibrionaceae	*Vibrio*	94,287	*Vibrio cholerae*	3362

The number of reads detected for each genus and for each species is listed. Reads for species do not add up to those of the genus because there are many reads that could be identified to genus but not to species.

**Table 2:** The mean number of reads of each bacterial genus from Table 1 for each bird species.

Bacterial genus	Mean number of reads in common reed warblers (# birds with reads)	Mean number of reads in lesser whitethroats (# birds with reads)	Mean number of reads in willow warblers (# birds with reads)
*Aeromonas*	335.0 (1)	NA (0)	1746.7 (3)
*Bacillus*	1454.0 (1)	1310.3 (3)	789.0 (3)
*Campylobacter*	5289.5 (2)	NA (0)	NA (0)
*Clostridium*	4798.0 (1)	4433.0 (1)	NA (0)
*Enterococcus*	29,409.7 (3)	6239.4 (5)	13,577.7 (3)
*Escherichia-Shigella*	3019.1 (8)	1563.5 (2)	4755.9 (7)
*Lactobacillus*	1240.0 (2)	5666.0 (2)	2023.0 (1)
*Listeria*	360.0 (1)	NA (0)	4153.0 (1)
*Mycoplasma*	2558.0 (1)	192,833.0 (1)	46,836.0 (1)
*Pseudomonas*	4486.1 (13)	5271.4 (12)	3112.1 (14)
*Ralstonia*	19,163.0 (20)	18,620.5 (20)	18,495.6 (19)
*Rickettsia*	16,238.0 (1)	NA (0)	646.0 (1)
*Serratia*	3759.4 (11)	2857.6 (12)	2949.8 (10)
*Staphylococcus*	22,305.0 (19)	6446.9 (17)	13,815.0 (16)
*Stenotrophomonas*	8202.4 (18)	5853.9 (18)	7400.6 (17)
*Streptococcus*	4224.4 (14)	2803.8 (9)	5588.9 (14)
*Vibrio*	NA (0)	12,009.0 (1)	41,139.0 (2)

We only used birds that were found to contain reads of each genus in calculating the mean. Numbers in parentheses denote the number of birds containing reads.

## Discussion

Our goal was to compare the gut microbiomes of three migrating passerines stopping over in Jordan during spring migration. We used 16S sequencing to characterize the bacterial diversity and composition of common reed warblers, lesser whitethroats, and willow warblers. We found that lesser whitethroats had more variation in their microbiomes than the other two bird species. Greater variation was seen in genus richness, Shannon diversity, and species composition, measured by NMDS, although richness was not significant. Lesser whitethroats also had the highest number of unique ASVs compared to the other two. The difference in variation may be due to their habitat preferences and food resources.

Willow warblers and reed warblers appear to have less variation in their habitat use during stopovers and winter range than lesser whitethroats, which may explain in part the differences in microbial diversity variation. When stopping over in Jordan, willow warblers are often recorded in dense scrub and foraging in open habitats with scattered shrubs, dwarf shrubs, and often in dense annual vegetation if available (pers. obs., FK). They are typical foliage gleaners and are often seen foraging in trees and bushes. Unlike in the breeding season, and unlike most other warblers during winter, which forage solitarily and show more sedentary behavior, the willow warbler tends to forage in groups, sometimes in mixed flocks, and it also tends to move from one area to another (“itinerancy” and low site persistence), moving away from areas that get dry to greener areas that contain more arthropods (Salewski et al. 2002; Lerche-Jørgensen et al. 2017; Thorup et al. 2019). Reed warblers that either migrate or breed in Jordan belong mainly to the eastern subspecies *fuscus*. In Jordan, migrant reed warblers prefer reeds and dense vegetation near water when stopping over (pers. obs., FK). Reed warblers are foliage gleaners but may also feed on arthropods on the ground or catch their prey aerially.

Lesser whitethroats stopping over in Jordan, on the other hand, are very flexible in the use of habitats, which usually include any type of scrub in arid and semi-arid areas, and woodlands, including gardens and plantations. They are usually the most abundant and frequent migratory warbler stopping over in desert scrub and in open woodlands (pers. obs., FK). Lesser whitethroats breed in woodlands, parks, and gardens in suburban and urban areas whenever suitable conifers and deciduous bushes are available, also young conifer plantations, vineyards, and orchards. In the core wintering areas in Eastern Africa, lesser whitethroats are abundant in open savanna with stands of *Tamarix, Balanites, Salvadora*, and *Acacia* with intermediate density (Wilson and Cresswell 2006), often along valleys and ravines, and on the edge of the desert and in oases (Helbig et al. 2010; Zwarts et al. 2016). Their site fidelity during winter is relatively low, and they forage solitarily. Their diet consists almost exclusively of arthropods, mostly small, soft-bodied insects, myriapods, spiders. Adults are occasionally frugivorous, taking small berries from many plant species and also nectar and pollen from flowers. Lesser whitethroats forage almost exclusively by gleaning leaves of bushes and trees (Helbig et al. 2010). This flexibility in habitat selection and their solitary foraging behavior, may account for some of the increased variation seen in the microbiomes of lesser whitethroats compared to willow warblers and reed warblers. Another factor could be that the birds in Jordan originate from various regions of Eurasia, including west-European populations. In the future, we plan to compare samples collected from the same species in Jordan, Georgia, and Ukraine. By comparing their microbiomes, we may be able to determine the origin of these species, and we can also determine how microbiomes change during the act of migration.

We found several potentially pathogenic genera in the three bird species. Environmental contamination by migratory birds in Jordan may be a risk to livestock and humans where birds stop and forage during their migration to their wintering grounds in sub-Saharan Africa (Reed et al. 2003; Smith et al. 2020, 2022). From the genera detected in these samples, we searched for reads that could be identified to species. Of particular interest are the following species and genera: *Ralstonia insidiosa, Ralstonia pickettii, Stenotrophomonas, V. cholerae, C. perfringens, Campylobacter*, and *Escherichia-Shigella*.

Both *R. insidiosa* and *R. pickettii* are emerging pathogens in hospital settings and have been found in many different types of water sources, including municipal drinking water and hospital water supplies (Ryan and Adley 2013). They are known to cause sepsis, with *R. pickettii* being associated with meningitis (Ryan and Adley 2013). Regarding *Stenotrophomonas*, there were only 3047 reads out of 378,826 that were identified as *Stenotrophomonas rhizophila*. Many *Stenotrophomonas* reads could not be identified below genus. One member of this genus is *Stenotrophomonas maltophilia* and is intrinsically multi-drug resistant. It is a concern in hospital settings and is regarded as an emerging global opportunistic pathogen. *Stenotrophomonas maltophilia* and *S. rhizophila* are closely related, which is most likely why 16S sequencing was not able to resolve most of the reads to species level for this group.

*Vibrio cholerae, C. perfringens, Campylobacter*, and *Escherichia-Shigella* are enteric pathogens and can cause severe diarrhea in humans. These pathogens cause hundreds of thousands of deaths each year around the world (Havelaar et al. 2015). *Campylobacter* is the most prevalent enteric pathogen in birds of the western US, and certain species are more associated with feedlots and crops, increasing potential for contamination and human infection (Smith et al. 2022). Two reed warblers were infected with *Campylobacter*; some reads could be identified to *Campylobacter gracilis*, but many could not be identified below genus. Likewise, it is unclear which species of *Escherichia-Shigella* are found in our samples, since they could not be identified below genus. Knowing which bird species contain pathogens of human importance and how the migration process could impact pathogen shedding at stopover sites should be a priority to assess instances of enteric pathogen introductions (Hubálek 2004; Mancuso et al. 2022).

There were no patterns regarding pathogenic genera and bird species. Most taxa were found in at least one individual of each bird species. Exceptions are *Aeromonas, Campylobacter, Clostridium, Listeria, Rickettsia*, and *Vibrio*. For example, one reed warbler and one willow warbler had reads from the genus *Rickettsia*. These ASVs could not be resolved to species, except for 646 that were identified as *Rickettsia* endosymbiont. It is unclear which species of *Rickettsia* were found in these two birds. Similarly, other pathogenic genera could not be identified to species, so we do not know which species of bacteria are present in these samples. These three bird species are likely to be similar in terms of pathogen shedding at stopover sites. Contamination of food crops and the environment could have impacts on livestock and human health (Smith et al. 2022). Wildlife management and conservation practices may be able to help limit where birds are foraging and defecating while at stopover sites. For example, crops can be planted far away from livestock feed lots, and deterrence methods may limit livestock–bird interactions (Smith et al. 2022).

The V3-V4 region of the 16S gene generally cannot resolve reads below genus, especially genera containing closely related species (Plummer et al. 2015). However, some regions are better than others (Bukin et al. 2019; Fadeev et al. 2021; López-Aladid et al. 2023). The V3-V4 region of the 16S gene, generally does a poor job compared to other regions at resolving reads below genus, especially genera containing closely related species (Fadeev et al. 2021; López-Aladid et al. 2023). Because of the limitations with V3-V4 16S sequencing, we do not know the species of many of the pathogenic genera that we selected. Some species could be determined, such as *C. gracilis, C. perfringens, E. cecorum, R. insidiosa, R. pickettii*, and *V. cholerae*, but the vast majority could not. There are many species/strains within each of these genera that are not known pathogens of birds. If the goal is to identify bacteria pathogens in animal or environmental samples to species or strain level, we suggest using shotgun metagenomics. Although much more expensive and resource intensive, this method sequences all the DNA present in a sample. It is not amplicon based, so it is agnostic in what is ultimately sequenced. This method is also less standardized for making comparisons among bacterial communities (alpha diversity, species composition), but it has the added benefit of detecting and characterizing gene functions, including those involved in antimicrobial resistance, which are important to document in the context of human and livestock health.

Our work shows differences in the diversity and composition of the bacterial microbiomes of three migrating passerines captured at a stopover site in Jordan during spring migration. Lesser whitethroats had more variation in bacterial richness, diversity, and composition than reed warblers and willow warblers. This may be due to their habitat flexibility or where they originated from. We found several genera and species that are pathogenic to humans, including *R. insidiosa* and *R. pickettii, V. cholerae, C. perfringens, Stenotrophomonas, Rickettsia*, and *Escherichia-Shigella*. We did not find any patterns regarding these pathogenic taxa and the three bird species. Many 16S reads could not be identified to species level, and we suggest using shotgun metagenomics to further characterize these pathogens, including identifying antimicrobial resistance genes. Migratory birds passing through Jordan may be a risk to livestock and humans through environmental contamination and contamination of food crops. Future work will seek to understand how migration impacts microbiome by sampling the same species in their breeding grounds and at stopover sites in Jordan. Wildlife management may help in limiting environmental contamination and reducing the risk of pathogens to livestock and humans.

## Authors contributions

N.H.: Conceptualization, Funding acquisition, Investigation, Project administration, Resources, Supervision, Writing—original draft, Writing—review & editing. M.R.: Conceptualization, Investigation, Methodology (Capturing and sampling birds, and 16S microbiome analysis), Writing—original draft, Writing—review & editing. F.K.: Conceptualization, Investigation, Methodology (Capturing, identifying, and ringing birds, and taking measurements), Writing—original draft, Writing—review & editing. A.A.: Methodology (DNA extraction for the birds’ fecal samples). A.H.: Methodology (Capturing and sampling birds) and Writing—original draft. G.Z.: Writing—original draft. A.I.: Methodology (Capturing and helping in identifying birds and taking measurements), Writing—original draft. A.B.: Conceptualization, Investigation, Methodology (Analysis of 16S microbiome data), Writing—original draft. C.E.: Methodology (analyzing the microbiome data), created figures, and wrote the results section. J.O.: Conceptualization, Funding acquisition, Writing—original draft, Writing—review & editing. J.F.: Conceptualization, Funding acquisition, Writing—original draft, Writing—review & editing.

## Supplementary Material

icaf015_Supplemental_File

## Data Availability

The 16S microbiome Sequence Read Archive (SRA) is available in the NCBI platform (https://www.ncbi.nlm.nih.gov/sra/PRJNA1236739) and referenced as PRJNA1236739 (accession numbers: SAMN47402528 to SAMN47402586). The data underlying this article are available within its context and in its supplementary material.
